# CITN11-02 interim trial results: subcutaneous administration of recombinant human IL-15 (rhIL-15) is associated with expansion of peripheral blood CD56+ NK cells and CD8+ T cells

**DOI:** 10.1186/2051-1426-3-S2-P203

**Published:** 2015-11-04

**Authors:** Chihiro Morishima, Douglas G McNeel, Manish R Patel, Holbrook E Kohrt, Thomas A Waldmann, John A Thompson, Kevin C Conlon, Paul M Sondel, Heather A Wakelee, Minjun C Apodaca, Steven P Fling, Mary L Disis, Stephen P Creekmore, Jeffrey S Miller

**Affiliations:** 1 University of Washington, Seattle, WA, USA; 2 Department of Medicine, University of Wisconsin-Madison, Madison, WI, USA; 3 University of Minnesota, Minneapolis, MN, USA; 4 Stanford University, Stanford, CA, USA; 5 National Cancer Institute, National Institutes of Health, Bethesda, MD, USA; 6 Department of Human Oncology, Department of Pediatrics, University of Wisconsin-Madison, Madison, WI, USA; 7 Fred Hutchinson Cancer Research Center, Seattle, WA, USA

## Background

The Cancer Immunotherapy Trials Network is conducting a Phase I, dose-escalation study of subcutaneous (SQ) rhIL-15 in advanced melanoma, renal cell, non-small cell lung (NSCLC) and squamous cell head and neck carcinoma patients. IL-15 has been identified as a high priority agent for immunotherapy development because it is a homeostatic factor for both NK cells and CD8+ T cells and, unlike IL-2, has little effect on suppressive regulatory T cells.

## Methods

Each cycle consists of 5 daily SQ injections of rhIL-15 (E.coli-derived, NCI) administered Monday-Friday for two weeks, followed by 2 weeks observation. The absolute lymphocyte count is tested every injection day, and whole blood flow cytometric analysis of T and NK cell frequencies is conducted on Days 1, 11 and 15 of each cycle.

## Results

Three patients have been enrolled in each of the 0.25, 0.5, 1.0 and 3.0 mcg/kg dose cohorts and six at 2.0 mcg/kg (N=18). Fourteen patients completed ≥2 cycles, three completed one cycle, and one patient (3 mcg/kg) received only 2 doses due to dose limiting toxicity (DLT). This last patient had NSCLC and developed grade 3 chest pain, hypoxia and dyspnea leading to hospitalization and discontinuation of rhIL-15. One serious adverse event, grade 2 pancreatitis, was observed in a metastatic melanoma patient 3 days after completing Cycle 1 (at 2.0 mcg/kg). Flow cytometric data indicate a consistent increase in the frequency of CD56+CD3-NK cell frequencies peaking at Day 15 of Cycle 1 (3 days after the last dose), with lesser increases in subsequent cycles. The mean fold-increase in circulating NK cells during Cycle 1 was 2.3, 3.3, 4.4, 9.6, and 11.8-fold for the 0.25, 0.5, 1.0, 2.0 and 3.0 mcg/kg dose cohorts respectively, demonstrating dose responsiveness. By contrast, the mean fold-increase in circulating CD8+ T cells was relatively modest at 1.1, 0.9, 1.2, 3.3, and 3.2-fold for the 0.25, 0.5, 1.0, 2.0 and 3.0 mcg/kg dose cohorts respectively.

## Conclusions

SQ rhIL-15 was well tolerated through 2.0 mcg/kg/dose and may be the maximum tolerated dose. One of 3 patients treated with 3.0 mcg/kg rhIL-15 experienced a DLT; this cohort will be expanded. Higher doses of rhIL-15 were associated with profound increases in circulating NK cells with smaller but still significant increases in CD8+ T cells. Outpatient use of subcutaneous rhIL-15 is safe and will likely emerge as a key agent for combination with other cancer immunotherapies.

Study support: NIH 1U01 CA154967-01

## Trial registration

ClinicalTrials.gov identifier NCT01727076.

